# Interspecific variation in persistence of buried weed seeds follows trade‐offs among physiological, chemical, and physical seed defenses

**DOI:** 10.1002/ece3.2415

**Published:** 2016-09-05

**Authors:** Adam S. Davis, Xianhui Fu, Brian J. Schutte, Mark A. Berhow, James W. Dalling

**Affiliations:** ^1^ USDA‐ARS Global Change and Photosynthesis Research Unit Urbana IL USA; ^2^ Department of Natural Resources and Environmental Sciences University of Illinois Urbana IL USA; ^3^ New Mexico State University Las Cruces NM USA; ^4^ USDA‐ARS National Center for Agricultural Utilization Research Peoria IL USA; ^5^ Department of Plant Biology University of Illinois Urbana IL USA

**Keywords:** chemical defense, dormancy, phenolic compounds, seed coat strength, seed defense syndromes, seed half‐life, seed trait covariance, soil seed bank, weed management, weed seedbank ecology

## Abstract

1. Soil seedbanks drive infestations of annual weeds, yet weed management focuses largely on seedling mortality. As weed seedbanks increasingly become reservoirs of herbicide resistance, species‐specific seedbank management approaches will be essential to weed control. However, the development of seedbank management strategies can only develop from an understanding of how seed traits affect persistence.
2. We quantified interspecific trade‐offs among physiological, chemical, and physical traits of weed seeds and their persistence in the soil seedbank in a common garden study. Seeds of 11 annual weed species were buried in Savoy, IL, from 2007 through 2012. Seedling recruitment was measured weekly and seed viability measured annually. Seed physiological (dormancy), chemical (phenolic compound diversity and concentration; invertebrate toxicity), and physical traits (seed coat mass, thickness, and rupture resistance) were measured.
3. Seed half‐life in the soil (*t*_0.5_) showed strong interspecific variation (*F*_10,30_ = 15, *p *< .0001), ranging from 0.25 years (*Bassia scoparia*) to 2.22 years (*Abutilon theophrasti*). Modeling covariances among seed traits and seedbank persistence quantified support for two putative defense syndromes (physiological–chemical and physical–chemical) and highlighted the central role of seed dormancy in controlling seed persistence.
4. A quantitative comparison between our results and other published work indicated that weed seed dormancy and seedbank persistence are linked across diverse environments and agroecosystems. Moreover, among seedbank‐forming early successional plant species, relative investment in chemical and physical seed defense varies with seedbank persistence.
5. *Synthesis and applications*. Strong covariance among weed seed traits and persistence in the soil seedbank indicates potential for seedbank management practices tailored to specific weed species. In particular, species with high *t*_0.5_ values tend to invest less in chemical defenses. This makes them highly vulnerable to physical harvest weed seed control strategies, with small amounts of damage resulting in their full decay.

## 1 Introduction

A large body of theory has been developed to advance our understanding of how aboveground plant life stages are defended, but little theory exists for the defense of seeds in the soil seedbank (Dalling, Davis, Schutte, & Arnold, 2011). For annual arable weeds, this is a critical knowledge gap, as their elasticity of population growth rate to seed survival in the soil seedbank is unity (Davis, 2006). Given the relatively recent, rapid proliferation of herbicide‐resistant genotypes (Powles & Yu, 2010), whose preferential survival allows them to contribute disproportionately to the replenishment of weed seedbanks, it is especially important to improve our understanding of intrinsic, seed‐based regulation of weed seed survival so that we may develop better management strategies targeted at the ecology of individual weed species (Gibson et al., 2016; Long et al., 2015).

In Dalling et al. (2011), we introduced a nascent framework for a seed defense theory to guide future investigations of intrinsic seed defense traits related to seed persistence in the soil seedbank. At its core is the concept of “seed defense syndromes” related to variation in seed dormancy types. Seeds of species with physical dormancy should be protected primarily by physical seed traits and rely upon rapid germination to escape pathogens. Seeds with physiological dormancy should be protected by a mixture of physical and chemical seed traits. Those species whose seeds are quiescent (nondormant and remain permeable over time) should rely on a mixture of chemical seed traits and mutualisms with microbial endophytes.

Annual weed species represent a class of plants whose dependence upon adaptations for seed survival in the soil seedbank is extreme. All members of the population must pass through the seed stage at some point, yet mortality in any given year is likely to be high for both seeds and seedlings. Weed management tactics typically target seedlings, with mortality rates commonly ranging between 90 and 99% for herbicide applications; tactics aimed at seeds are possible (Walsh, Newman, & Powles, 2013) but rare. Weed seeds also face numerous environmental hazards. They are an important food source for many small vertebrates and invertebrates (Westerman, Borza, Andjelkovic, Liebman, & Danielson, 2008), with pre‐ and postdispersal granivory ranging from 30% to 90% of total annual seed production (Davis, Daedlow, Schutte, & Westerman, 2011). Tillage practices that move weed seeds below their maximum germination depth and stimulate germination at the wrong time of year and conditions favoring pathogenic fungi all increase seed mortality to fatal germination (Benvenuti, Macchia, & Miele, 2001; Davis & Renner, 2007). The importance of seed decay due to microbial attack varies by species, environment, and burial depth, with annual losses reaching 50% (Davis et al., 2005).

Exposed to such high levels of uncertainty and risk in their growing environment, annual weeds have evolved highly variable, complex forms of seed dormancy that include physical dormancy, innate dormancy, induced dormancy, conditional dormancy, deep and nondeep physiological dormancy, and subannual dormancy cycling, among others (Baskin & Baskin, 2001). Indeed, in annual cropping systems, seed dormancy appears to be a fundamental signature of weediness. For example, *Bassia scoparia* [L.] A. J. Scott (kochia) is a weed of the northern great plains of the USA that has historically been nondormant, or had very low levels of dormancy (Zorner, Zimdahl, & Schweizer, 1984). However, under increased intensity of weed management, dormancy levels in *B. scoparia* have risen steadily as has its weediness (Esser, 2014).

Weed communities tend to be species poor compared with early successional natural communities, often composed of one or two dominant species and 20 or less subdominant species. However, even the relatively short list of common weed species harbors considerable diversity in seed traits (Gardarin & Colbach, 2015), presumably because ongoing, stochastic change in agricultural management practices and climate maintains the adaptive value of divergent seed characteristics by creating an ever‐changing composition of niches in the soil seedbank. Seeds of annual weed species vary greatly in size, shape, mass, chemical composition, cohort size, seed dormancy type and level, dormancy‐breaking cues, maximum emergence depth, and recruitment cues, among numerous other dimensions (Gardarin & Colbach, 2015; Long et al., 2015). Thus, not only do weed seedbanks represent an important but underutilized management target, but also they are particularly well suited for investigating the relationship between seed persistence and seed traits.

Our aim was to determine the level of empirical support for the existence of seed defense syndromes (Dalling et al., 2011) among annual weeds of arable lands. Experimental objectives were framed by the hypothesis that seedbank persistence covaries with seed traits of arable weeds, such that the balance of physiological, chemical, and physical seed defenses varies among weed species with different half‐lives in the soil seed bank. Our experimental objectives were to (1) quantify long‐term persistence of 11 seedbank annual weed species in a common environment, (2) characterize their physiological, chemical, and physical seed traits, and (3) relate findings of this study to previous work through a broad quantitative comparison.

We found that while physiological, chemical, and physical seed traits all contribute to seed persistence in the soil seedbank, physiology (seed dormancy) is a primary driver of seed persistence. Covariances among seed traits offered some support for the theory of seed defense syndromes comprised of suites of traits. Quantitative comparisons of our results to other published work indicated that weed seed dormancy underlies seed persistence across a broad range of weed species and growing environments and that early successional species' relative investment in chemical and physical seed defenses depends strongly on their level of persistence in the soil seedbank.

## 2 Materials and Methods

### 2.1 Burial study

**Figure 1:**
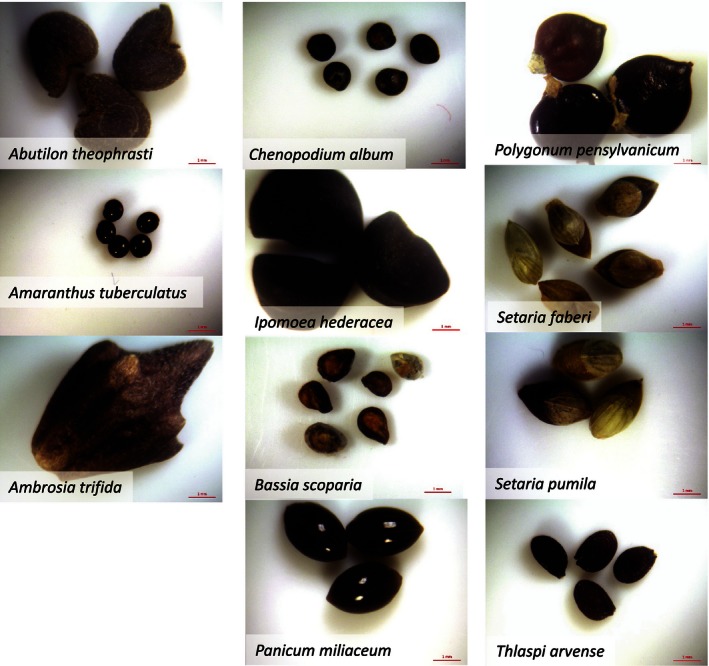
Seeds of 11 arable weed species included in the common garden study

We performed a common garden weed seed burial study at the University of Illinois Crop Sciences Research and Education Center in Savoy, IL (40.048757 N, −88.237206 E), from October 2007 through October 2012. The experiment was arranged in a split‐plot design with four replications of the subplot variable *species* nested within main plot variable *burial duration* (1–5 years). Eleven annual weed species were included, spanning a broad range of seed sizes, dormancy types, and seedbank persistence (Table 1, Table S1, Fig. 1): *Abutilon theophrasti* Medik (velvetleaf), *Ambrosia trifida* L. (giant ragweed), *Amaranthus tuberculatus* [Moq]. Sauer (common waterhemp), *Bassia scoparia* [L.] A. J. Scott (kochia), *Chenopodium album* L., *Ipomoea hederacea* Jacq. (ivyleaf morningglory), *Panicum miliaceum* L. (wild proso millet), *Polygonum pensylvanicum* L. (Pennsylvania smartweed), *Setaria faberi* Herrm. (giant foxtail), *Setaria pumila* [Poir] Roem. (yellow foxtail), and *Thlaspi arvense* L. (field pennycress).

**Table 1:** Seed fate of eleven annual arable weeds in common garden study of soil seedbank persistence

Species name	Dormancy type^a^	Seed fate (%)^b^			Half‐life (years)
		Dormancy	Germination	Mortality	
*Abutilon theophrasti*	Physical	77 (3.4)	11 (1.4)	13 (3.8)	2.22 (0.25)
*Ambrosia trifida*	ND physiological	32 (6.4)	19 (4.0)	49 (7.8)	0.60 (0.17)
*Amaranthus tuberculatus*	D/ND physiological	67 (3.6)	8.0 (1.7)	25 (4.4)	1.65 (0.13)
*Bassia scoparia*	ND physiological	5.1 (4.9)	6.3 (3.0)	89 (5.6)	0.25 (0.00)
*Chenopodium album*	CD/ND physiological	69 (5.6)	9.5 (3.3)	22 (5.0)	1.94 (0.43)
*Ipomoea hederacea*	Physical	41 (6.4)	24 (5.6)	35 (7.3)	0.82 (0.09)
*Panicum miliaceum*	D/ND physiological	39 (8.1)	17 (5.4)	45 (8.8)	1.16 (0.22)
*Polygonum pensylvanicum*	ND physiological	34 (6.6)	24 (6.5)	42 (7.3)	0.43 (0.02)
*Setaria faberi*	ND physiological	33 (6.0)	34 (6.5)	34 (8.2)	1.00 (0.23)
*Setaria pumila*	CD/ND physiological	25 (7.6)	13 (6.8)	62 (9.0)	0.50 (0.10)
*Thlaspi arvense*	D/ND physiological	4.1 (2.2)	22 (7.8)	74 (8.4)	0.36 (0.07)

a As reported in Baskin and Baskin (2001). Dormancy status abbreviations for species with physiological dormancy are as follows, where “/” represents dormancy cycling: ND, nondeep; D, deep; CD, conditionally dormant.

b Seed fate and half‐life values represent means (±SE) over a 5‐year burial period for four replicate blocks of the field study.

Weed seeds were collected in 2007 from the experimental site and adjoining fields by gently shaking mature inflorescences over a bucket and bulking seeds from multiple plants to form a composite sample for each species (i.e., a single biological replicate). Light seeds were removed by processing with a seed cleaner, after which seeds were stored in airtight containers at 4C until burial. Immediately prior to burial, seed viability was assayed with tetrazolium (Peters, 2000). Burial units consisted of 100 seeds of a given species placed in the bottom of a 2.5‐cm‐deep square tray, 10 cm on a side, made of 0.5‐mm stainless steel wire mesh. Tray bottoms were permeable to water, but prevented seeds from escaping. Trays were filled 2 cm deep with soil from a nearby grass sward that had not been cropped for over 30 years, to avoid contamination with weed seeds (verified by elutriating samples of this soil). Within each experimental unit, we excavated a 2‐cm‐deep rectangle 30 cm wide by 40 cm long and placed trays for each of the 11 species side by side into this depression so that their soil surface was flush with the surrounding soil, leaving a 0.5‐cm wire mesh lip exposed in each tray. Each experimental unit was covered by wire mesh with 1‐cm square openings to permit access to invertebrate granivores. The study plot was fenced to exclude large vertebrates.

Seedling emergence was recorded weekly from March through October every year. Seed trays for a given *burial duration* treatment were removed in October of the assigned year and seeds recovered via elutriation through sieves with a 0.5‐mm mesh (Wiles, Barlin, Schweizer, Duke, & Whitt, 1996). Recovered seeds were incubated under oscillating temperature conditions (15 C/dark for 10 hr, 25 C/light for 14 hr) for 2 weeks and germination recorded. Ungerminated seeds assessed as viable (alive) through tetrazolium testing (AOSA 2000) were considered dormant.

Seed fate classes for year *t *+* *1 were calculated with respect to viable seeds (*N*_sd_) present in October of year *t* and included

(1) $$\text{persistence}=\frac{N_{\mathrm{sdl}}\left(t+1\right)+N_{\mathrm{sd}}\left(t+1\right)}{N_{\mathrm{sd}}\left(t\right)}$$

(2) $$\text{mortality}=\frac{N_{\mathrm{sd}}\left(t\right)-\left[N_{\mathrm{sdl}}\left(t+1\right)+N_{\mathrm{sd}}\left(t+1\right)\right]}{N_{\mathrm{sd}}\left(t\right)}$$

(3) $$\text{germination}=\frac{N_{\mathrm{sdl}}\left(t+1\right)}{N_{\mathrm{sd}}\left(t\right)}$$

(4) $$\text{dormancy}=\frac{N_{\mathrm{sd}}\left(t+1\right)}{N_{\mathrm{sd}}\left(t\right)}$$

where persistence + mortality = 1 and *N*_sdl_ = seedling emergence. By this classification, dormant seeds include only viable seeds that remain ungerminated at time *t* + 1, whereas persistent seeds include all viable seeds at time *t* + 1 (comprised of those that germinated and gave rise to seedlings and those that remained ungerminated). For some of the smaller‐seeded species, fatal germination may have occurred from the 2.5 cm depth, increasing the amount of mortality recorded, and decreasing the amount of germination recorded.

### 2.2 Seed traits

We measured chemical and physical seed traits on freshly collected seeds following the methods outlined in Tiansawat, Davis, Berhow, Zalamea, and Dalling (2014), using multiple measures of each trait class to provide functional redundancy and allow them to be treated as latent or manifest variables during multivariate analyses (Hatcher, 1994). For the chemical defense trait class, we measured *ortho*‐dihydroxyphenol (*o*‐DHP) concentration (Hendry & Grime, 1993), abundance, and diversity of phenolic compounds quantified with high‐performance liquid chromatography (Gallagher et al., 2010), impact of seed homogenate on brine shrimp survival (Lieberman, 1999), and seed removal by invertebrate granivores (Davis et al., 2011). Physical traits measured included seed coat thickness, seed mass, and seed coat rupture force (Text S1). Pairwise interspecific phylogenetic distances were quantified using the *phydist* subroutine of Phylocom 4.2 (www.phylodiversity.net). Detailed methods for characterizing seed chemical and physical traits and phylogeny are presented in online supplemental information for this article (Text S1, Fig. S1).

### 2.3 Data analysis

We analyzed seed persistence time series data with nonlinear mixed‐effects models, relating viable seeds remaining over time to an asymptotic exponential model (Pinheiro & Bates, 2004) that included burial duration as a fixed effect and block nested within species as a random effect (Table S2). The decay function was expressed as

(5) $$y=\text{Asym}+\left(R_{0}-\text{Asym}\right)*e^{-e^{lrc*x}}$$

where *y* = persistent seeds remaining, Asym = horizontal asymptote, *R*_0_ = y‐intercept, lrc = natural log of the exponential rate constant, and *x *= burial duration (years). We calculated half‐lives for seed persistence in the soil seedbank (*t*_0.5,_ the time for half the seeds in the population to exit the soil seedbank, through either mortality or germination) from the replicate‐level model, where *t*_0.5_ = ln(2)/exp(lrc).

Mixed‐effects models were also used to quantitatively compare our results with published data, following the approach of Miguez, Villamil, Long, and Bollero (2008). Our comparison did not reach the level of a full meta‐analysis because our data requirements for the comparison (studies had to include weed germination, viability, and seed persistence data across three or more years for multiple species or genotypes) limited acceptable studies found in our search of online literature databases. We found five additional studies for the analysis of dormancy versus seed persistence (Buhler & Hartzler, 2001; Conn, Beattie, & Blanchard, 2006; Gleichsner & Appleby, 1989; Liebman et al., 2014; Roberts & Feast, 1972). For the analysis of relative investment in chemical and physical defenses with variation in seed half‐life, we found two additional studies (Davis, Schutte, Iannuzzi, & Renner, 2008; Tiansawat et al., 2014), focusing on arable weeds and tropical pioneer trees, respectively. All mixed‐effects models were analyzed in the *nlme* package of R 3.2.3 (R Foundation for Statistical Computing, Vienna).

Covariance modeling of associations among seed traits and seed persistence included factor analysis and structural equation modeling (Hatcher, 1994) for tests of seed defense syndromes, partial least squares regression (Carrascal, Galvan, & Gordo, 2009) for identifying phenolic compounds related to *t*_0.5_, and simple and partial correlations for quantifying contributions of seed traits and phylogeny to variation in *t*_0.5_. Factor analysis models included physical and chemical seed traits as manifest variables and were fit using varimax rotation with the minimum number of factors determined through analysis of scree plots and chi‐squared tests. Structural equation models were selected by maximum likelihood from a candidate pool of models that all included *t*_0.5_ as a common endogenous variable and varied in the number of seed trait classes (physiological, chemical, and physical) and variable types (manifest or latent) that were included as exogenous variables (Table S3). Partial least squares regression models included *t*_0.5_ as a common dependent variable and included phenolic compound presence and abundance as independent variables. Multivariate analyses were performed in the *psych*,* plsr, lavaan*, and *corpcor* packages of R 3.2.3.

## 3 Results

### 3.1 Seed fate

**Figure 2:**
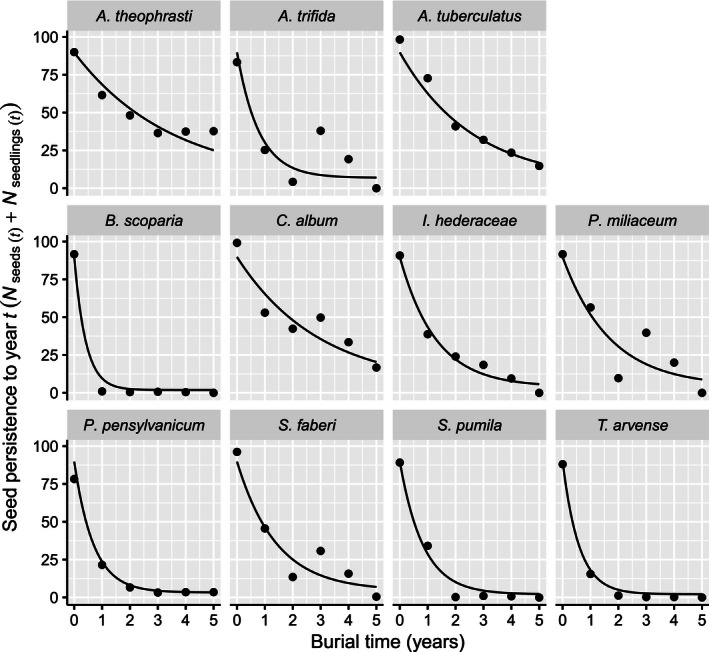
Seed persistence in the soil seedbank, over a 5‐year burial period, for seeds of eleven annual arable weed species. Lines represent within‐group predictions from an asymptotic exponential nonlinear mixed‐effects model. Points represent the means of four replicate blocks of seed viability data per species per year

Seedbank decline was smooth for seven species (*A. theophrasti*,* A. tuberculatus*,* B. scoparia*,* I. hederaceae*,* P. pensylvanicum*,* S. pumila*, and *T. arvense*) and showed little variation among blocks, whereas seedbank decline of the remaining species (*A. trifida*,* C. album*,* P. miliaceum* and *S. faberi*) was considerably more variable (Fig. 2). Percent viability of the initial seed lots varied, as determined by tetrazolium staining, and therefore, the number of persistent seeds at t_0_ in Figure 1 was not 100 for all species. Also, because we cleaned our starting populations of seeds to remove light seeds, the populations were likely enriched in viable seeds compared with a natural population. After 5 years of burial, fewer than 50% of the original viable seeds persisted for all 11 species, and fewer than 25% persisted for all species except *A. theophrasti*.

Seed persistence time series data were fit well by a single nonlinear mixed‐effects model (for clarity, mean values are shown in Fig. 1; replicate‐level fits are shown in Fig. S2). The most parsimonious model contained random effects for all three parameters of the asymptotic exponential model but no fixed effect for *species* (Table S2). A linear mixed‐effects model of *t*_0.5_ indicated a strong main effect of *species* (*F*_10,30_ = 15, *p *< .0001). Seed *t*_0.5_ ranged from 0.25 years (*B. scoparia*) to 2.22 years (*A. theophrasti*) (Table 1). Six of the species had *t*_0.5_ values <1 year, four had *t*_0.5_ values between 1 and 2, and only one had a *t*_0.5_ value >2.

Analysis of mean seed fates across years showed strong effects of *species* on dormancy (*F*_10,180_ = 19, *p *< .0001), germination (*F*_10,180_ = 19, *p *< .0001), and mortality (*F*_10,180_ = 14, *p *< .0001). Seed fate fell into three distinct classes, by species: (1) species with mean dormancy >65% (*A. theophrasti*,* A. tuberculatus*, and *C. album*), (2) species with mean dormancy between 25% and 45% (*A. trifida*,* I. hederaceae*,* P. miliaceum*,* P. pensylvanicum*,* S. faberi*, and *S. pumila*), and 3) species with mean dormancy of 5% or less (*B. scoparia* and *T. arvense*). Across weed species, *t*_0.5_ was strongly correlated with dormancy (*r *= .94, *p *< .0001) and mortality (*r *= −.87, *p *< .001), but not with germination (*r *= −.38, *p *= .22). Seed germination rate was not correlated across species with either dormancy (*r *= −.34, *p *= .28) or mortality (*r *= .006, *p *= .98), but dormancy and mortality were strongly negatively correlated (*r *= −.94, *p *< .0001).

An important caveat for interpreting these results is that our approach to quantifying seedbank persistence, in which seeds were unprotected and allowed to exit the seedbank through germination, or various types of mortality, led to measurements of dormancy that differ from those made in seed persistence studies in which buried seeds are protected by a container of some sort. In this study, dormancy calculations were limited to those seeds that had survived in a given annual time period and did not include those seeds that had been killed by pathogens or granivores. This aspect of the experimental design likely means that those seeds with greater defenses against seed predation or decay were also likely to be classified as being more dormant, as reflected in the structural equation model results reported in the next section.

### 3.2 Covariances among seed traits and persistence

Seed chemical and physical traits varied widely among species (Table S1). Seed mass ranged over two orders of magnitude from *A. tuberculatus* and *C. album* (0.022 and 0.039 g per 10^2^ seeds, respectively) to *A. trifida* and *I. hederacea* (4.53 and 2.76 g per 10^2^ seeds, respectively). Other physical seed traits were correlated with seed mass, including seed coat rupture force (*r *= .62, *p *= .03) and seed coat thickness (*r *= .50, *p *= .09). Direct measures of seed defense chemistry were highly correlated among themselves. Seed concentration of *o*‐DHP was correlated with both total number of phenolic compounds (*r *= .88, *p *< .001) and total concentration of phenolic compounds (*r *= .89, *p *< .001), which were correlated with each other (*r *= .92, *p *< .0001). None of these putative measures of chemical defense investment were significantly correlated with a functional assessment of the toxicity of seed homogenates to invertebrates, as represented by a brine shrimp rearing assay (*p *> .15 in all cases). However, seed chemical defense level, measured as the total concentration of phenolic compounds, was strongly negatively correlated with seed palatability to invertebrate granivores (*r *= −.70, *p *= .01), measured through field predation trials adjacent to the common garden burial location (Fig. S3).

Factor analysis model selection indicated that a model with two factors was best supported by the data (Table 2). Factor 1 explained 34% of the variance in seed traits, and contained positive loadings for physical (seed coat rupture force, seed coat thickness, and seed mass) and chemical (phenolic peak area, number of phenolic peaks, and *o*‐DHP concentration) seed traits. Factor 2 explained 21% of the variance in seed traits and contained positive loadings for chemical seed traits (number of peaks and invertebrate toxicity) and a negative loading for physiological seed traits (seed dormancy). Communality scores (h^2^), a measure of the amount of variation explained for individual variables in the model, were relatively high, with six of nine scores >0.50.

**Table 2:** Factor analysis of physiological, chemical, and physical seed traits of annual arable weed species in seed burial study

Seed trait	Factor 1^a^	Factor 2	h^2^ b
Dormancy (%)		−0.56	0.31
Seed coat rupture force (N)	0.62		0.39
Seed coat thickness (μm)	0.86		0.79
Seed mass (mg)	0.58		0.54
Seed coat ratio^c^			0.51
Phenolic peak area (Abs/mg)	0.78		0.67
Phenolic compounds (number of peaks)	0.66	0.83	0.71
o‐DHP concentration (μg g seed^−1^)	0.67		0.46
Invertebrate toxicity^c^		0.75	0.59
Variance explained (%)	34	21	
Cumulative variance explained (%)	34	55	

a Only factor loadings ≥0.5 were retained.

b h^2^ = communality, the amount of variation explained for individual variables.

c Seed coat ratio, seed coat thickness/seed mass; invertebrate toxicity, brine shrimp ED_50._

Because the richness of phenolic compounds (total peak number) was important to both factors 1 and 2, we conducted a partial least squares regression to determine whether the presence of certain phenolic peaks, and their concentrations, showed associations with *t*_0.5_ across weed species. A three component model explained 33% of the variation in *t*_0.5_, with 69.2%, 21.8%, and 5.4% of the variation explained by components 1 through 3, respectively (Table S4). Peak presence or absence did not contribute loadings to any of these variables, but the concentrations of individual peaks did. Component 1 showed a strong negative relationships between *t*_0.5_ and total phenolic peak area (loading = −0.92) plus peak area of putative defense compounds *a* (loading = −0.49; retention time = 14–14.5 s) and *b* (loading = −0.24; retention time = 21.5–22 s). Components 2 and 3 showed loadings for peak areas of 8 other putative defense compounds. No other variables were retained in the PLS analysis.

Structural equation models were then used to quantify putative causal associations among individual seed traits and *t*_0.5_ while accounting for covariances among these traits. Although the global model contained variables for physiological, chemical, and physical seed traits, model selection strongly indicated the central importance of seed dormancy in determining *t*_0.5_ (Table S3). The most parsimonious SEM model retained only the exogenous variable dormancy (Model 10, *b*_1_ = 0.85, *p *< .0001, Akaike weight = 0.99) and explained 73% of the variation in *t*_0.5_. The second best supported model retained only the exogenous variable Factor 2 (Model 13, *b*_1_ = 0−.70, *p *< .0001, Akaike weight = 0.01) and explained 49% of the variation in *t*_0.5_. Latent variables for physical traits had positive associations with *t*_0.5_ in SEM models (*b*_1_ = 0.41, *p *< .05), whereas latent variables for chemical traits had negative associations with *t*_0.5_ (*b*_1_ = −0.79, *p *< .01).

Finally, to determine whether associations among seed traits and *t*_0.5_ were a by‐product of evolutionary relatedness, we conducted partial correlations of seed traits and *t*_0.5_ while accounting for phylogenetic distance (Table S5). Only a weak phylogenetic signal was detected, with no partial correlations of *t*_0.5_ and phylogenetic distance >−0.13, and correlations between *t*_0.5_ and dormancy or F_2_ remaining significant even when partialling out phylogenetic distance.

### 3.3 Quantitative comparison with other studies

We quantitatively compared our results to previously published results for (1) the strong positive association between dormancy and seed half‐life in the soil seedbank and (2) the trade‐offs among physical and chemical defenses in relation to *t*_0.5_ as seen in the factor analysis and SEM results. In the latter quantitative comparison, we addressed potential trade‐offs among physical and chemical defenses as systematic variation in the relative investment in chemical defense (represented by total phenolic concentration) and physical defense (seed coat thickness) in response to variation in t_50_ among different species.

**Figure 3:**
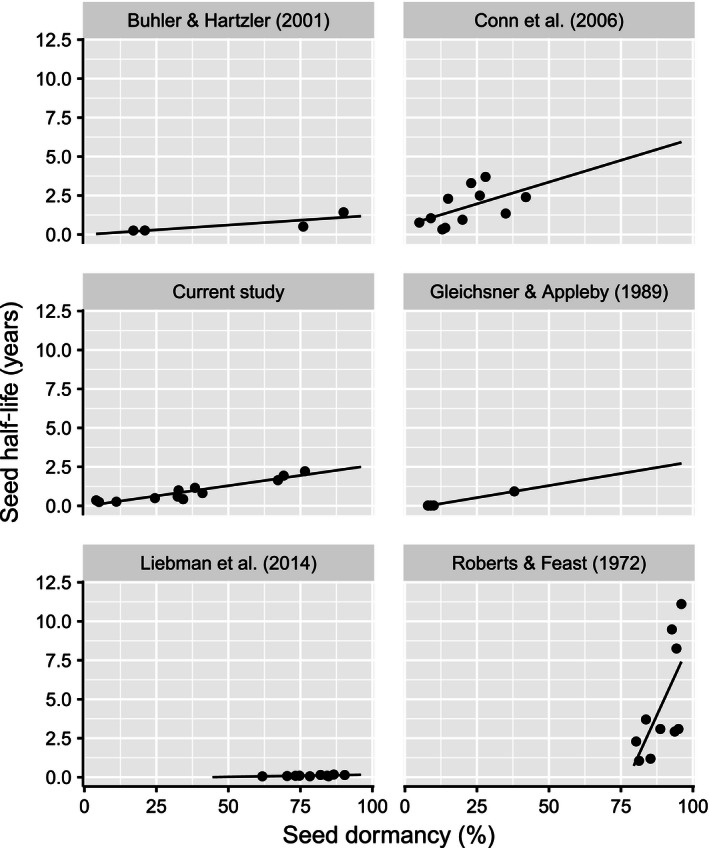
Seed half‐life in the soil seedbank in relation to seed dormancy for the current study and five previously published studies of weed seed persistence in the soil seedbank. Lines represent within‐group predictions from a linear mixed‐effects model, and points represent mean values for seed half‐life and seed dormancy

The relationship between *t*_0.5_ and mean seed dormancy for all six studies, including the current study, was best described by a linear mixed‐effects model with a fixed effect for the interaction between dormancy and study and random shifts to intercept by study (Fig. 3). Although slopes varied greatly among studies, with a particularly steep slope for the data from Roberts and Feast (1972), the relationship between mean seed dormancy and *t*_0.5_ remained linear and positive for all weed seed persistence studies included in the comparison.

**Figure 4:**
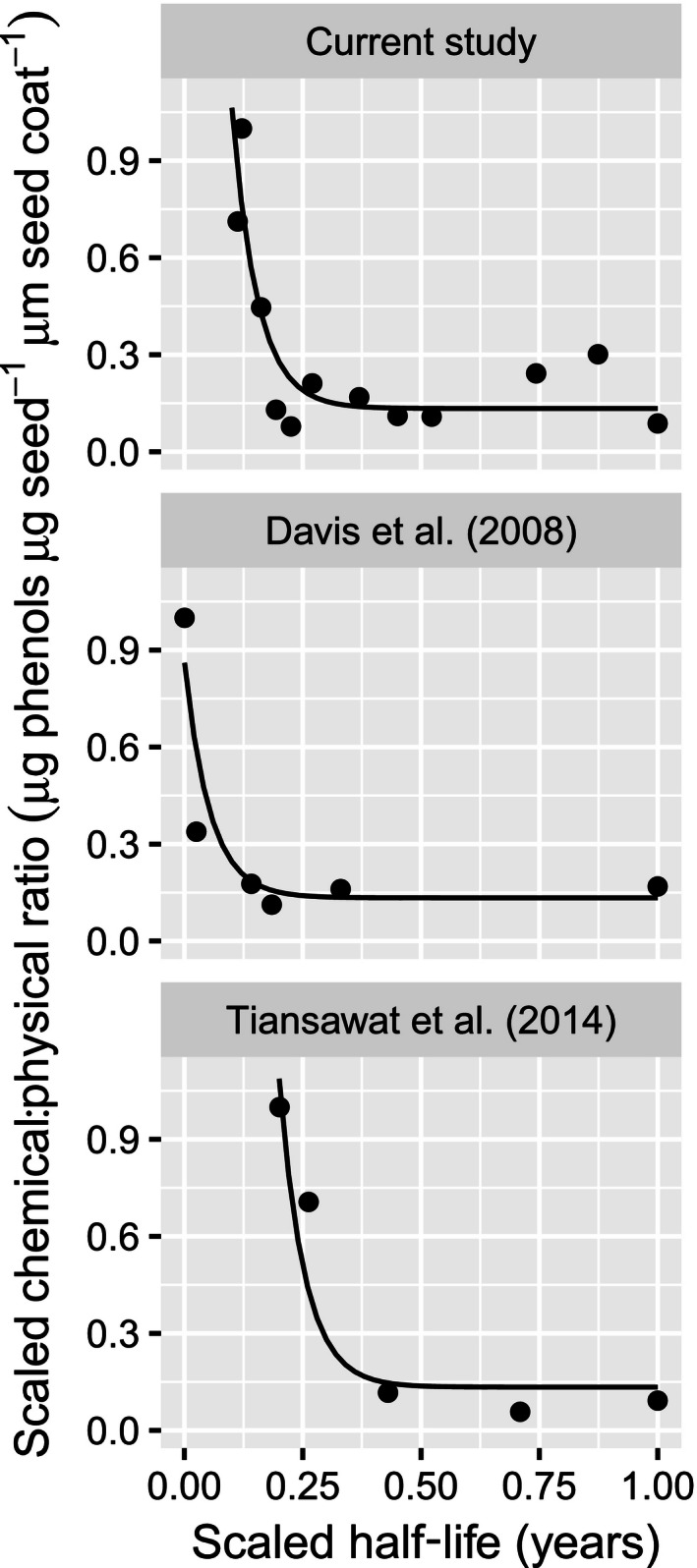
Trade‐off between chemical and physical defense (ratio of total phenolic concentration in seed homogenate to seed coat thickness) in relation to variation in seedbank persistence for the current study and two previously published studies. Independent and dependent variables were rescaled to a range of 0–1. Lines represent within‐group predictions from an asymptotic exponential nonlinear mixed‐effects model with “study” treated as a random factor and half‐life treated as a fixed factor

Relative investment in chemical and physical seed defenses, in relation to variation in *t*_0.5_ among early successional species, was best described by a nonlinear mixed‐effects model using an asymptotic exponential function and a random effects structure that included random shifts to each of the model parameters, by study (Fig. 4). For all three studies, investment in chemical defenses, relative to physical defenses, was greatest for species with lowest persistence in the soil seedbank and declined rapidly with increasing values of *t*_0.5_.

## 4 Discussion

Our results supported the hypothesis that seedbank persistence covaries with seed traits of arable weeds, with varying emphasis on physiological, chemical, and physical seed defenses among weed species with different *t*_0.5_. Following the terminology of Long et al. (2015), species *t*_0.5_ was proportional to various types of “resistance” to exiting the soil seedbank, including resistance to seed losses though germination (dormancy) and mortality (chemical and physical defenses; our study did not include measures of seed aging). As quantified through factor analysis and SEM, species with varying *t*_0.5_ solved the problem of persistence through contrasting approaches: Species with longer *t*_0.5_ relied more on dormancy and physical defense for persistence, whereas species with shorter *t*_0.5_ relied less on dormancy and more on chemical defense for persistence. These results corroborate those of Gardarin and Colbach (2015), who found that associations among physical and chemical seed traits and dormancy explained as much as 56% of variability in seed dormancy, and extend upon them by relating these traits to seedbank persistence.

Our results offered somewhat more tentative support for the specific seed defense syndromes proposed in Dalling et al. (2011). Due to the limitations of the species we were working with (i.e., arable weed seeds of temperate agroecosystems, none of which are quiescent to our knowledge), we were only able to examine two of the three syndromes (physical–chemical and physiological–chemical). Seed traits segregated into two factors aligned with these syndromes (Table 2), indicating that seed traits may covary in such a way as to represent syndromes. However, a stronger test of this hypothesis must wait for future studies in which multiple populations of various species are compared in several common garden environments to determine the relative importance of intrinsic seed traits and environmental context in shaping resultant suites of seed characteristics related to persistence in the soil seedbank. Another factor to consider in such future studies is evidence of positive associations between seed coat permeability and seed coat phenolic compounds in certain crop species (Mohamed‐Yasseen, Barringer, Splittstoesser, & Costanza, 1994), which indicates a potential correlation between chemical defenses and physical dormancy. Therefore, an additional experimental design feature that would improve the ability to disentangle these defense traits in weed seeds would be to include time series data on seed permeability and dormancy in relation to the chemical defense profile of the study seeds.

Comparing results from the current study with previously published data indicated that the covariances among seed traits and seedbank persistence quantified here are fundamental features of weed seedbanks across a broader range of weed species and agroecosystems. The strong positive association between weed seed dormancy and persistence in the soil seedbank has been long recognized by weed scientists and perceived as an organizing principle for weed management strategies (Chepil, 1946; Gardarin & Colbach, 2015; Schafer & Chilcote, 1969).

The physical and chemical seed defenses studied here have been found by others to have functional significance in protecting against microbial decay (Gallagher et al., 2010) and seed predation (Lundgren & Rosentrater, 2007). Of 30 putative phenolic defense compounds, 10 were related to *t*_0.5_. The negative association observed between total phenolic concentration and seed predation of study species by invertebrates in adjacent plots (Fig. S3) offers one possible mechanism whereby variation in seed traits could have translated into variation in *t*_0.5_, as seed trays were accessible to invertebrate granivores.

### 4.1 Management implications

We observed rates of weed seedbank decline that were similar to rates reported for shallow burial studies (Buhler & Hartzler, 2001), but considerably faster than rates reported in studies where weed seeds were broadcast and incorporated into soil through tillage (Lutman et al., 2002; Roberts & Feast, 1972) or buried within closed vessels (Burnside, Wilson, Weisberg, & Hubbard, 1996; Telewski & Zeevaart, 2002). For the most persistent species (*A. theophrasti*,* A. tuberculatus*, and *C. album*), values of *t*_0.5_ were an order of magnitude lower than in studies where the same species were buried more deeply (Davis et al., 2008). Seed resistance to exiting the soil seedbank, either through germination or mortality, increases with burial depth (Long et al., 2015). Weed seedbanks in this study likely declined quickly for all species because seeds were maintained within 2 cm of the soil surface and therefore exposed to a full range of mortality factors as well as germination cues. All weed seed half‐lives in this study were <2.5 years, underscoring opportunities for proactive seedbank management.

Producers will benefit from considering the potential for different species to form more or less persistent seedbanks when weighing contrasting seedbank management options. Harvest weed seed control, an approach whose adoption is currently somewhat limited due to cost constraints, can destroy a large proportion of newly produced weed seeds (Walsh et al., 2013). The reduced investment in chemical defenses, relative to physical defenses, for longer‐lived weed species observed in this and other studies (Fig. 3) makes them an especially attractive target for harvest weed seed control. In such species, low levels of chemical defense make them particularly vulnerable to physical damage such as that resulting from mechanical harvest weed seed control tactics as only very small amounts of damage to the seed coat can result in full seed decay when such seeds enter the soil seedbank (Davis et al., 2008).

When weed communities are dominated by species with low *t*_0.5_, or a producer is considering switching from a high to low soil disturbance management system, seedbank management through tillage may be an appropriate option. Deep tillage can temporarily reduce seedling emergence from a large seed production cohort by placing the bulk of the seed population below maximum germination depth (DeVore, Norsworthy, & Brye, 2012); however, repeated tillage will bring deeply buried seeds back to the surface again. Therefore, this should be considered a rescue strategy, rather than an annual seedbank management practice, for species with highly persistent seeds and especially for herbicide‐resistant weed genotypes.

Finally, the central importance of weed seed dormancy in regulating seedbank persistence underscores the need for additional management tactics to exploit this vulnerability. Quantifying genetic and epigenetic contributions to seed dormancy (Chao, Dogramaci, Anderson, Foley, & Horvath, 2014; Gu, Kianian, & Foley, 2006) remains an important knowledge gap to be addressed as a means of identifying particularly persistent populations and also to determine whether in‐field environments may be manipulated to reduce weed seed dormancy (Nurse & DiTommaso, 2005). The efficacy of existing strategies for depleting the soil seedbank through seedling emergence, such as superficial soil disturbance to stimulate seed germination (“stale seedbeds”), may be enhanced by fine‐tuning them to species‐specific dormancy‐breaking requirements through multigenotype, multi‐environment studies (Long et al., 2015; Schutte & Davis, 2014; Schutte et al., 2014).

## Conflict of Interest

None declared.

## Data Accessibility

The data analyzed in this publication are hosted at USDA AgData Commons as the following data resource: Davis, Adam (2016) Data from: Interspecific variation in persistence of buried weed seeds follows trade‐offs among physiological, chemical, and physical seed defenses. Ag Data Commons: http://dx.doi.org/10.15482/USDA.ADC/1288753.

## Funding Information

Funding for this work was provided by USDA‐ARS (ASD, MAB).
