# Supplementary material for: Interspecific variation in persistence of buried weed seeds follows trade‐offs among physiological, chemical, and physical seed defenses
Source: Ecol Evol. 2016 Sep 5;6(19):6836–45. doi: 10.1002/ece3.2415 (PMC5513235; doi:10.1002/ece3.2415)
Supplement: Supplementary file 1 [file ECE3-6-6836-s001.docx]

**Text S1**. Extended methods for characterization of seed traits.

CHEMICAL TRAITS

Seed chemical defences can consist of many different types of compounds, depending upon the plant species (Kremer, 1986; Guimaraes *et al.*, 2003; Veldman *et al.*, 2007). We focused on the characterization of phenolic compounds, as they have been found to be important components of weed seed defences in other studies (Kremer, 1986; Hendry *et al.*, 1994; Davis *et al.*, 2008; Gallagher *et al.*, 2010). Seed phenolic chemistry was assayed both as a broad chemical group (*ortho*-dihydroxyphenols; *o*-DHP) through colorimetric methods (Hendry & Grime, 1993) and as a collection of disparate individual compounds by quantification with high powered liquid chromatography (HPLC).

To assess seed *o*-DHP content using the colorimetric approach of Hendry and Grime (1993), we first ground whole seeds to a fine homogenate using a Wiley mini-mill (Thomas Scientific, Swedesboro, New Jersey, USA) fitted with a 0.5 mm screen. In between processing different weed seed accessions, we passed a sample of *Zea mays* L. seed through the mill to clean out seed residue from its interior. Seed homogenates were then extracted with methanol, centrifuging at 12,300 xg for 2 minutes, after which 250 μL of the supernatant was transferred to a spectrophotometric cuvette with 250 μL of 100 mM Tris-HCl buffer (pH 6.7) and 1 mL 100 mM KTi oxalate. Samples from weed seed extracts were run through a Beckman Spec-20 spectrophotometer (Beckman Coulter Inc., Indianapolis, Indiana, USA) at a 445 nm wavelength. Sample *o*-DHP content was then calculated by comparing sample absorbance to a standard curve of reagent grade pyrogallol made in 50 nM increments from 0 to 250 nM.

HPLC measurements of weed seed total phenol content were made, following the methods of Gallagher et al (2010) in analytical laboratories at the USDA-ARS National Center for Agricultural Utilization Research in Peoria, Illinois, USA. To ensure that we obtained a comprehensive profile of phenolic compounds contained in seed homogenates, we extracted volatile and nonvolatile fractions (Gallagher *et al.*, 2010; Granger *et al.*, 2011). Seed sample homogenates were defatted with hexane in individual scintillation vials, sonicating for 60 minutes, letting stand overnight, and sonicating again for 60 minutes. The hexane layer (volatile fraction) was then removed to an HPLC vial for analysis. The remaining defatted material was allowed to air dry in a fume hood for 24 hours to remove all hexane. Three 0.1g aliquots of defatted seed homogenate were then weighed out into separate scintillation vials and 1.5 mL methanol added to each vial. Vials containing the seed-methanol mixture were sonicated for two 60 minute periods, as described above, and then 1 to 1.5 mL supernatant filtered through a hypodermic syringe filter (0.45 um pore, nylon 66 matrix) into a HPLC vial (soluble fraction). To obtain phenols in the bound fraction of the seed homogenate, remaining supernatant was removed from the scintillation vials and the pellet hydrolyzed with 5 mL of 4 N NaOH, the vial sealed and incubated at 60 C for 12 hours. Following hydrolysis, samples were brought to a pH of 2 by adding approximately 1.5 mL of concentrated HCl. Samples were then dried under N_2_ gas for 48 hours until completely dry, the contents resuspended in 1.5 mL of methanol and finally transferred to an HPLC vial for quantification of bound phenols.

Seed extracts were analysed for phenolic compounds with a Shimadzu LC-10A HPLC system (two LC-10AT pumps, SIL-10A autosampler, a CTO-10A column oven, a SPD 10AVi variable wavelength UV-Vis detector and a SCL-10Avp system controller, running under Shimadzu LCSolutions version 1.25 chromatography software, Columbia, MD, USA). The column used was an Inertsil ODS-3 reverse phase C-18 column (5 µM, 250 x 4.6 mm, with a Varian metaguard column). Initial conditions for phenolic compound analysis were 20% methanol and 80% water with 0.05 M phosphoric acid, at a flow rate of 1 ml minute^-1^. The effluent was monitored at 280 and 340 nm on the VWD. After injection (25 µL), the column was held at the initial conditions for two minutes, then developed to 100% methanol in a linear gradient over 55 minutes. Peak detection was at 280 nm (see Fig. S1 for sample chromatogram). Standards of a variety of phenolics were run at 15 uLs per injection of a 1 mg/mL solution. Mass-standardised peak areas were calculated as

$\frac{\frac{Raw peak area}{Injection volume}x Total volume of extract}{Sample mass}$ [1]

where peak area was measured in mV x min, injection volume was 25 μL, extract volume was 1.5 mL, and sample mass was measured in g.

Following sample analysis with HPLC, putative phenolic peaks were edited using a three step algorithm. First, all peaks with retention times less than 45 seconds were removed. Next, all peaks with standardised areas greater than 2 x 10^5^ absorbance units g sample^-1^ were removed. Finally, neighboring peaks with retention time differences less than 0.5 minutes were binned and treated as the same putative phenolic compound. We distinguished among phenolic defence compounds using retention times, and compared relative concentrations of these compounds among different species using mass-standardised peak areas (Tiansawat *et al.*, 2014).

Functional significance of seed defence chemistry was assessed both directly, through toxicity of seed extracts to brine shrimp (Lieberman, 1999), and indirectly through relative palatability of seeds to invertebrate granivores in short-duration, mid-summer seed removal assays in maize plots adjacent to the burial units, as was done in Davis et al. (2011). Brine shrimp were hatched in covered test tubes containing aqueous solutions with different concentrations of seed extract. Seed homogenates were de-fatted, as described above, extracted with methanol and then allowed to dry in a fume hood. The remaining pellet was re-suspended in water and added to different test tubes, in various volumes, in combination with a prepared salt-water aquarium mixture, to create a range of concentrations of seed extract. Seed extract concentrations included 0 μg mL^-1^ (control), 10 μg mL^-1^, 100 μg mL^-1^, 1 mg mL^-1^, 5 mg mL^-1^ and mg mL^-1^ and were included in four replicate blocks of the assay.

PHYSICAL TRAITS

Seed weights were quantified with an analytical balance precise to ± 0.0001 g. Seed coat thickness was measured to the nearest μm for 10 seeds of each species with a dissecting microscope. Seeds were cut in half, seed coat thickness measured at three random locations and measurements recorded with image analysis data acquisition software. Seed coat rupture force was measured using an Instron 5900 electromechanical force testing system (Instron, Norwood Massachusetts, USA) with a force transducer capable of recording rupture forces between 0 and 500 N. Ten seeds of each species were tested with the Instron to quantify mean seed coat rupture force.

**References for extended methods**

Davis, A. S., Daedlow, D., Schutte, B. & Westerman, P. R. (2011) Temporal scaling of episodic point estimates of weed seed predation to long-term predation rates. *Methods in Ecology and Evolution,* **2,** 682-692.

Davis, A. S., Schutte, B. J., Iannuzzi, J. & Renner, K. A. (2008) Chemical and physical defense of weed seeds in relation to soil seedbank persistence. *Weed Science,* **56,** 676-684.

Gallagher, R. S., Ananth, R., Granger, K., Bradley, B., Anderson, J. V. & Fuerst, E. P. (2010) Phenolic and short-chained aliphatic organic acid constituents of wild oat (*Avena fatua* L.) seeds. *J Agric Food Chem,* **58,** 218-225.

Granger, K. L., Gallagher, R. S., Fuerst, E. P. & Alldredge, J. R. (2011) Comparison of seed phenolic extraction and assay methods. *Methods in Ecology and Evolution,* **2,** 691-698.

Guimaraes, P. R., Jose, J., Galetti, M. & Trigo, J. R. (2003) Quinolizidine alkaloids in *Ormosia arborea* seeds inhibit predation but not hoarding by agoutis (*Dasyprocta leporina*). *Journal of Chemical Ecology,* **29,** 1065-1072.

Hendry, G. A. F. & Grime, J. P. (1993) *Methods in comparative plant ecology*. Chapman & Hall, London. pp. 252

Hendry, G. A. F., Thompson, K., Moss, C. J., Edwards, E. & Thorpe, P. C. (1994) Seed persistence - a correlation between seed longevity in the soil and *ortho*-dihydroxyphenol concentration. *Functional Ecology,* **8,** 658-664.

Kremer, R. J. (1986) Antimicrobial activity of velvetleaf (*Abutilon theophrasti*) seeds. *Weed Science,* **34,** 617-622.

Lieberman, M. (1999) A brine shrimp bioassay for measuring toxicity and remediation of chemicals. *Journal of Chemical Education,* **76,** 1689-1691.

Tiansawat, P., Davis, A. S., Berhow, M. A., Zalamea, P. C. & Dalling, J. W. (2014) Investment in Seed Physical Defence Is Associated with Species' Light Requirement for Regeneration and Seed Persistence: Evidence from Macaranga Species in Borneo. *PLOS ONE,* **9**.

Veldman, J. W., Murray, K. G., Hull, A. L., Garcia, J. M., Mungall, W. S., Rotman, G. B., Plosz, M. P. & McNamara, L. K. (2007) Chemical defense and the persistence of pioneer plant seeds in the soil of a tropical cloud forest. *Biotropica,* **39,** 87-93.

Table S1. Physical and chemical seed traits of eleven arable weed species in seedbank persistence study

Species Seed coat Seed coat Seed Seed Brine Seed [*o*-DHP] Number Total

rupture thickness mass coat shrimp ED_50_ predation (μg *o*-DHP of phenol phenolic

force (N) (μm) (g 10^-2^ sd) ratio* (μg mL^-1^) (% removed d^-1^) μg ^-1^ sd) peaks peak area

*A. theophrasti* 58 112 0.87 130 0.13 61 22.4 3 4.59

*A. trifida* 51 109 4.53 24 0.15 97 152.8 7 0.0028

*A. tuberculatus* 9.0 14 0.022 606 0.16 19 17.2 1 3.36

*B. scoparia* 12 28 0.11 265 0.10 78 34.1 6 4.88

*C. album* 37 44 0.039 1150 0.60 64 9.2 4 7.4

*I. hederaceae* 79 136 2.76 49 0.32 12 96.4 7 3.45

*P. miliaceum* 21 60 0.47 128 1.00 91 27.4 2 1.38

*P. pensylvanicum* 15 152 0.49 311 0.69 32 305.8 6 21.9

*S. faberi* 53 60 0.18 324 0.56 87 24.9 2 1.57

*S. pumila* 13 84 0.32 259 0.25 90 12.0 2 1.32

*T. arvense* 17 81 0.11 770 1.77 33 23.2 11 19.1

* Explanation of seed trait names: seed coat ratio = seed coat thickness/seed mass; brine shrimp ED_50_ = concentration of seed extract (μg mL^-1^) resulting in 50% survival of brine shrimp in bioassay; seed predation = mean % seeds eaten by invertebrate granivores in adjoining field plots in two 24 hour periods; [*o*-DHP] = *ortho*-dihydroxyphenol concentration in seed homogenate; number of phenol peaks = number of different peaks, per species, measured with HPLC; total phenolic peak area = sum of phenolic peaks, by species, measured with HPLC.

Table S2. Nonlinear mixed effects model of weed seed persistence in common garden study at Savoy, IL, 2007 through 2012

Model parameters*

**Fixed term** *Asym* *lrc* *R_0_*

Estimate 1.79 -0.26 90.1

**Random effects**

Burial duration (*sd*)† 0.02 0.77 0.002

Residual (*sd*) 11.3

* Data were fit to an exponential model with the following functional form

$$y= asym+\left( R_{0}-Asym \right)*e^{-e^{lrc*x}}$$

where *y* = viable seeds remaining, *asym* = the horizontal asymptote, *R_0_* = response when input is zero, *lrc* = natural log of the exponential rate constant and *x* = burial duration in years.

† sd = standard deviation

Table S3. Model selection for structural equation models of seed half-life in the soil seedbank (*t_0.5_*) in relation to chemical, physical and physiological seed traits

Model V_1_* V_2_ V_3_ cov_12_ cov_13_ cov_23_ b_1_ b_2_ b_3_ R^2^ k AIC *w_i_*

1 LV_chem_† LV_phys_ 0.72** -0.79** 0.41* 0.34 16 796 0

2 LV_chem_ -0.50** 0.27 8 466 0

3 dorm peaks force -0.48** 0.20 0.09 0.78*** -0.10** 0.04 0.74 10 437 0

4 dorm force 0.29 0.85*** 0.02 0.73 6 323 0

5 dorm peaks -0.48* 0.81*** -0.09 0.38 6 314 0

6 peaks force 0.09 -0.52*** 0.31* 0.34 6 365 0

7 F_2_ force -0.33* -0.69*** -0.03 0.50 6 348 0

8 F_1_ dorm 0.21 0.12 0.82*** 0.75 6 322 0

9 F_1_ F_2_ -0.62*** -0.24 -0.86*** 0.53 6 328 0

**10 dorm 0.85*** 0.73 3 197 0.99**

11 peaks -0.49*** 0.24 3 242 0

12 force 0.26 0.07 3 251 0

13 F_2_ -0.70*** 0.49 3 224 0.01

14 F_1_ 0.29* 0.11 3 250 0

* Explanation of model specification and model selection symbols: V_i_ = exogenous variables 1 through 3; cov_ij_ = covariances among exogenous variables 1 through 3; b_i_ = standardized regression coefficients between V_i_ and *t_0.5_* (seed half-life in soil seedbank, in years), with *, ** and *** representing significance levels of p< 0.05, 0.01 and 0.001, respectively; R^2^ = coefficient of determination (1-latent error for *t_0.5_*); k = number of free parameters in model; AIC = Akaike Information Criterion; *w_i_* = Akaike weight for the i^th^ model (probability that a given model is the most parsimonious of the candidate pool of models).

† Explanation of exogenous variable names: LV_chem_ = latent variable for seed chemical traits, comprised of number of phenolic peaks, total phenolic peak area, and o-DHP concentration (μg o-DHP μg seed homogenate^-1^); LV_phys_ = latent variable for seed physical traits, comprised of seed rupture force (N), seed coat thickness (μm) and seed coat ratio (seed coat thickness (μm)/seed mass(μg)); dorm = mean annual seed dormancy (%); peaks = number of phenolic peaks in seed homogenate of each species, as determined through HPLC; force = seed coat rupture force (N).

Table S4. Retention times and partial least squares regression (PLS) loadings for thirty five phenolic peaks detected in HPLC assay of seeds of eleven arable weeds

Peak Retention time Retention time Related to seed PLS Loading

ID lower (sec) upper (sec) half-life by PLS? C1 C2 C3

a 14 14.5 yes -0.49 0.92

b 21.5 22 yes -0.23 -0.70 0.96

c 16.5 16.5 yes -0.60

d 20 20.5

e 23.5 24 yes 0.15

f 11.5 11.5

g 12.5 12.5 yes -0.22

h 14.5 15 yes -0.33

i 15.5 16 yes -0.33

j 20.5 21

k 22.5 23 yes 0.12

l 24.5 25

m 31.5 32

n 34 34.5

p 10 10

q 13.5 14 yes -0.13

r 17 17.5

s 18 18 yes 0.29

t 19 19

u 23 23.5

v 25.3 25.6

w 27 27.7

x 28 28.5

y 29 29

z 30 30

aa 32 32.5

ab 33 33

ac 3.4 3.7

ad 9 9

ae 3 3

af 5 5.5

ag 6.5 6.5

ah 26 26.5

ai 31 31.5

aj 33.5 34

Table S5. Simple and partial correlations of pairwise distances among species for seed persistence, phylogeny and selected chemical and physical seed traits

Variables* Simple correlations† Partial correlations‡

a b c cor_a,b_ cor_a,c_ pcor_a,b|c_ pcor_a,c|b_

*t_0.5_* dormancy phydist 0.88*** -0.10 0.87*** -0.002

*t_0.5_* F_physiol.chem_ phydist 0.53*** -0.10 0.52*** -0.02

*t_0.5_* peaknum phydist 0.09 -0.10 0.12 -0.13

*t_0.5_* peak area phydist -0.16 -0.10 -0.17 -0.02

*t_0.5_* invert toxicity phydist -0.11 -0.10 -0.12 -0.11

*t_0.5_* rupture force phydist -0.004 -0.10 -0.01 -0.11

*t_0.5_* coat thickness phydist -0.02 -0.10 -0.02 -0.10

*t_0.5_* seed mass phydist -0.18 -0.10 -0.17 -0.09

*t_0.5_* coat ratio phydist 0.16 -0.10 0.15 -0.09

* Explanation of variable names for pairwise distances in: dormancy = mean annual seed dormancy (%) from seed persistence study; F_physiol.chem_ = factor 2 from factor analysis (dormancy, number of phenolic compounds, invertebrate toxicity); peaknum = number of phenolic peaks detected in chemical analysis of seed homogenate; peak area = total area of phenolic peaks detected in chemical analysis of seed homogenate; invert toxicity = brine shrimp ED_50_ in bioassay; rupture force = seed coat rupture force (N); coat thickness = seed coat thickness (μm); seed mass in (μg); coat ratio = coat thickness/seed mass.

† Pearson correlation between variable pair denoted by lower case letters.

‡ Partial correlation between variable pair denoted by lower case letters, partialling out the covariance with the variable indicated to the right of the vertical line.





Figure S1. Sample chromatogram from HPLC analysis of weed seed homogenate.

Figure S2. Replicate-level nonlinear mixed effects model of seed persistence


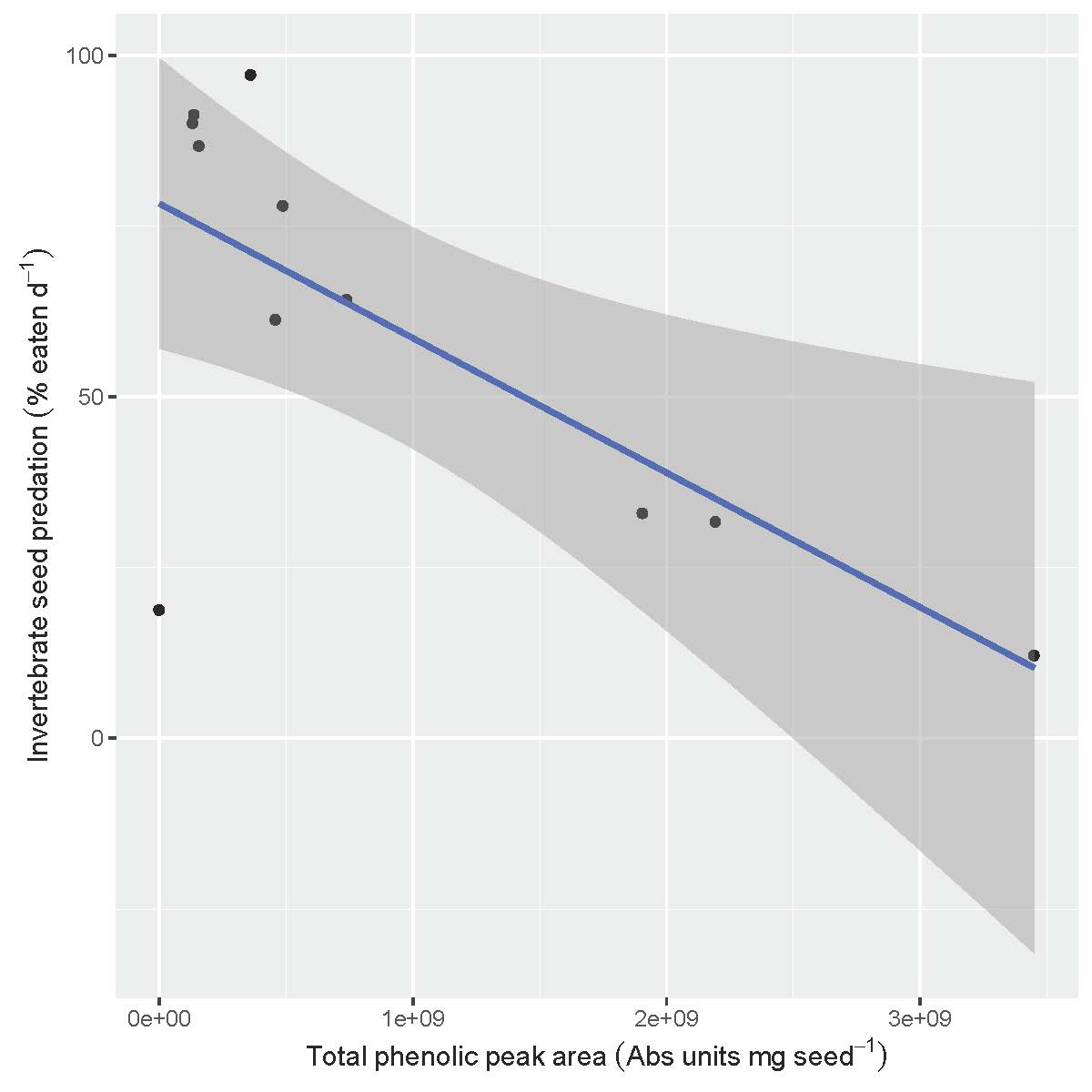


Figure S3. Invertebrate seed predation and chemical seed defence
